# Effects of Hospital Workers’ Friendship Networks on Job Stress

**DOI:** 10.1371/journal.pone.0149428

**Published:** 2016-02-22

**Authors:** Sung Yae Shin, Sang Gyu Lee

**Affiliations:** 1 Department of Quality Improvement and Patient Safety, Gangnam Severance Hospital, Yonsei University College of Medicine, Seoul, Korea; 2 Department of Hospital Management, Graduate School of Public Health, Yonsei University, Seoul, Korea; Örebro University, SWEDEN

## Abstract

**Background:**

This study attempted to identify the sources of job stress according to job position and investigate how friendship networks affect job stress.

**Methods:**

Questionnaires based on The Health Professions Stress Inventory (HPSI) developed by Wolfgang experienced by healthcare providers were collected from 420 nurses, doctors and radiological technologists in two general hospitals in Korea by a multistage cluster sampling method. Multiple regression analysis was used to examine the effects of friendship networks on job stress after controlling for other factors.

**Results:**

The severity of job stress differed according to level of job demands (p = .006); radiologic technologists experienced the least stress (45.4), nurses experienced moderate stress (52.4), and doctors experienced the most stress (53.6). Those with long-term friendships characterized by strong connections reported lower levels of stress than did those with weak ties to friends among nurses (1.3, p < .05) and radiological technologists (11.4, p < .01). The degree of cohesion among friends had a positive impact on the level of job stress experienced by nurses (8.2, p < .001) and radiological technologists (14.6, p < .1). Doctors who participated in workplace alumni meetings scored higher than those who did not. However, those who participated in alumni meetings outside the workplace showed the opposite tendency, scoring 9.4 (p < .05) lower than those who did not. The resources from their friendship network include both information and instrumental support. As most radiological technologists were male, their instrumental support positively affected their job stress (9.2, p < .05). Life information support was the primary positive contributor to control of nurses’ (4.1, p < .05), radiological technologists’ (8.0, p < .05) job stress.

**Conclusion:**

The strength and density of such friendship networks were related to job stress. Life information support from their friendship network was the primary positive contributor to control of job stress.

## Introduction

The social network effect on healthcare provider’s job stress has been studied less among both researchers and practitioners. Although the impact of social networks on individuals completing task [1–3], work performance [4–6], and promotion [7] at workplace is widely recognized. An understanding of how social networks and support influence the stressors can contribute to an insight of effective interventions for alleviating job stress. The provision of social support has concepts that explain the important functions of social networks that surround individuals. Thus, social network can be seen as linkages between people that may provide social support [8] that served as a protective intervention to people at high risk on the negative effects of stress [9, 10].

Especially, the job stress of healthcare providers has been recognized as a serious social problem [11]. Indeed, continued exposure to high levels of job stress is of concern not only because it involves ongoing personal suffering [12–15] but also because it may threaten the quality of patient care [16–19]. Taking into consideration workers’ health and improvement of the quality of care, the present study intends to contribute at the management level efforts to eliminate stress for all workers. Job stress for all jobs has sources and levels that can be measured and compared in the work conditions to give insight for reducing stress of each job.

In this paper, we focus primarily on network in which the ties refer to friendship and its social support. Specifically, the structure of friendship network measured by network strength, and density and network support has been measured by two types of supportive acts: instrumental support, informational support [20].

Many previous studies consistently find social network as a vital role to reduce the negative effect of stress [21–23] through two important functions; help-seeking [24] and psychological adjustment [25] at the workplace. To date, with regard to help seeking for reducing job stress, research has focused on organizational setting. For example, research done related to the social network of healthcare providers has just focused on management support and co-workers support [26–30]. Organizational changes can prevent or reduce stressors, but there are still several unique factors in the work condition that remain.

In a report in 2008, NIOSH (National Institute for Occupational Safety and Health) defined that job stressors are as the following: job demands, organizational settings, and individual settings: economic factors, conflict between work and family responsibilities [31]. In other words, job stress originates in demands and pressure from both within and outside of the workplace. In individual aspect, serious stress related to work-family conflicts [32] come from outside of the work. Further, NIOSH (1999) view that individual factors can help to reduce the effects of stressful working conditions includes a support of friends and coworkers [33]. Even though, these are largely tested on informal ties in organizational settings [34–38] job stress reduction research has not shown the impact of friendship networks (framed with people inside and outside the workplace).

Second, research on the effectiveness of social network for job stress has been published, but it fails to explain the specific mechanisms [39–41]. Since the late 1980s, however, sociologists have recognized that social networks influence an individual's psychological and physical health and have examined mechanisms as to how social network may have positive effects on health outcome [42–44]. For example, research results showed that people with many friends are not as susceptible to disease as those with a only a few friends [45, 46]. Also, breast cancer patients’ low death rate was associated with instrumental support (such as access to care) and physical health [47]. Research has shown that there is improvement of health and prevention of disease through mechanisms of social network. In the same context, the need was found to research friendship network to see its effectiveness of managing job stress through strengthening friendship networks and enhancing the exchange of social support.

The purpose of this study is: (1) to evaluate the sources and the level of job stress according to the healthcare provider (2) to determine the relationship between friendship networks and job stress and (3) to examine the mechanism how friendship network and social support affects job stress.

## Methods

This research was approved by Gangnam Severance Hospital, Institutional Review Board. (# 3-2015-0097).

### Subjects

This project involves nurses, doctors and radiological technologists who are positions with high levels of job stress [48, 49]. Radiological technologists (technologists) were included because among high levels of job stress positions that position had the largest number.

### Sampling frames

The data analyzed in this study was obtained in 2011 from workers under one foundation in two general hospitals that are both in Seoul, South Korea. One hospital has 2000 beds, and the other has 800 beds. The 800-bed hospital’s work environment provided 719 nurses, 517 doctors and 68 technologists and the multistage cluster sampling method was used. Being under the same foundation the organization settings are the same for both hospitals. Doctors can work three-month terms in each hospital; however, the doctors selected were doctors serving in the 800-bed hospital. The adequate number of nurses serving in the 800-bed hospital was selected for this project. The technologists were selected is an equal number from both hospitals. Even though the random sampling procedure from lists of elements is usually most ideal, the occupational socioeconomic status and work conditions vary so it was not practical for random sampling. Therefore, multistage cluster sampling method was highly efficient for this project [50]. Determination of sample size was calculated using the G power 3.19 program. At least 77subjects were necessary to provide sufficient power to detect a significant difference for effect size = 0.15, p = .05, and power = 0.8. In the case of nurses, 320 nurses were selected from the general section that has fifteen units (odd numbered units were selected equaling eight units) and the special section that has four units (one of each was selected equaling three units). Among 320 nurses, 299 questionnaires were received, and the number of 288 questionnaires were fully completed, so 288 were utilized in the project.

In the case of doctors, 80 doctors (physicians, residents and interns) were selected from the sixteen clinical-specialty groups. Brennan’s research (1991) in New York State hospital found that the rate of adverse events among clinical-specialty groups found a higher number in the surgical group compared to the non-surgical group [51]. The researcher postulated that job stress in the surgery group would be higher than the non-surgical group; therefore, 35 doctors were equally selected from both the surgical and non-surgical groups. There are eight sub-specialties in the surgery group where 35 doctors were selected from three sub-specialties. There are seven sub-specialties in the non-surgical group where 35 doctors were selected from internal medicine. Also, ten doctors were selected from emergency medicine group. Among 80 doctors, 68 questionnaires were received, and all 68 questionnaires were fully completed, so the number utilized in the project was 68.

In the case of technologists, there were 68 technologists at the 800-bed hospital and 136 technologists at the 2,000-bed hospital. 80 questionnaires were given technologists, 40 were given to technologists at each hospital. Among the 80 technologists, 73 questionnaires were received, and 64 questionnaires were fully completed, so the number utilized in the project was 64.

In short, 420 individuals were participated in the project (total return rate: 87.5%; return rate among nurses: 90%; return rate among doctors: 85%; return rate among technologists: 80%). The gender ratio of population and the gender ratio of this project is similar. The gender ratio of population (focused on female) is 96% of 719 nurses, 37% of 517 doctors and 19% of 204 technologists which is compared to the gender ratio of the project that is 93% of 288 nurses, 37% of 68 doctors and 17% of 64 technologists. Participation in the research study was voluntary, and a participant could drop out of the study at any time. Verbal consent was given by each participant. The questionnaire was deposited in a collection box.

### Measures

#### Dependent variable: Job stress

Inter-professional differences must be defined when approaching the management of job stress. The job-related stress inventory that compares the sources and levels of stress among different job positions of healthcare professionals is needed. The questionnaire for healthcare professionals was prepared by the Health Professions Stress Inventory (HPSI) developed by Wolfgang [52]. Wolfgang’s research (1988) focused on doctors, nurses and pharmacists, and nurses were found with the highest level of job stress [53], Korean nurses’ job stress came from work and patient factors [54]. Each item is scored from 0 to 4, and overall job stress is calculated by summing the ratings for all items. Scores range from 0 to 120, and higher scores indicate higher levels of stress. Participants were asked to complete the 30-item questionnaire, which assesses the levels and sources of job stress on a five-point scale ranging from “never” to “very often.” Self-administered questionnaires were used to collect data on job-related stress. The questionnaire (S1 Text) has shown to have Cronbach’s α coefficient 0.93.

#### Independent variables: Friendship network

Previous studies were examined to see that three dimensions of friendship networks: strength, and density. Measurements of friendship net and network support followed the major concepts and definitions established by Cohen, Underwood and Gottlieb [55]. Strength stands for daily contact and high density stands for mutual acquaintance between friends and family. Within this strength and high-density network there is practical support and various information flows. This study defined a close friend as a “person who comfortably borrows money from you or with whom you spend time on birthdays or holidays” [56]. We employed the following definitions of the two dimensions: (1) The strength of friendship networks was measured in terms of the duration of the friendship and the frequency of meetings. Types of friend included friends from work, friends from college, and friends from pre-college schools (high school, middle school, and elementary school); we assumed that these fell on a continuum from short term to long term. Additionally, a strong friendship was defined as one that involved, on average, at least, one daily meeting, and a weak friendship was defined as one that involved, on average, fewer than one daily meeting [45]. (2) The density of friendship networks was measured in terms of whether a friend in a network knew other friends in that network (knew at least one friend = 1, did not know anyone = 0). Additionally, we included two variables to address group activities in and support from friendship networks: whether respondents participated in group meetings, such as alumni association meetings restricted to co-workers and held at the workplace, and alumni association meetings open to non-employees and held outside the workplace (attendance at least once a year = 1, less than once a year = 0). Finally, the kinds of support obtained from friendship networks were examined to include life information, instrumental information (tangible aid and services), and job vacancies information. A variety of these support categories can be given by the same friend.

### Analyses

#### Descriptive statistics

The population-level characteristics of participants were analyzed using one-way ANOVA test. Job stress influenced by gender [57, 58], age [59], marital status [60], education, household income, and friendship network and support variables were examined and statistics provided for each job position.

#### Sources of job stress

Factor analysis was performed for sources of stressors. Based on the results, we formed seven factors from the 30 questions (Table 1) which are based on rotated factor patterns: (1) decision-making authority, (2) conflicts with co-workers, (3) role/work overload, (4) conflicts with patients, (5) receiving respect from the patients, (6) job advancement, and (7) work-family conflicts. Seven factors were separated into three dimensions: organizational setting (factors 1, 2), job demands (factors 3–5) and individual setting (factors 6, 7) to examine the differences among job positions. One-way ANOVA test was performed to examine the mean stress scores and factors to compare the differences.

**Table 1:** Rotated factor patterns related to job stress.

Category	Item	Factor						
		1	2	3	4	5	6	7
Decision-making authority	Not being allowed to participate in making decisions	.780	.184	.070	.228	.187	.077	.167
	Not being able to use abilities to the fullest extent on the job	.723	.056	.077	.173	.216	.082	.246
	Not receiving feedback on job performance	.712	.178	.193	.193	.174	-.004	.103
	Experiencing conflicts with supervisors	.689	.374	.174	.087	-.003	.182	-.195
	Not knowing what type of job performance is expected	.619	.276	.315	-.006	.150	.112	-.194
	Supervising the performance of coworkers	.577	.386	.146	.086	.066	.177	-.329
Conflicts with co-workers	Experiencing conflicts with co-workers	.131	.766	.133	.192	.133	.042	.027
	Not having opportunities to share feelings with co-workers	.213	.746	-.050	.047	.017	.016	.043
	Having non-health professionals determine the way you must practice your profession	.192	.695	.277	.116	.102	.136	-.053
	Possessing inadequate information regarding a patient’s medical condition	.165	.653	.074	.062	.111	-.071	.163
	Not being recognized or accepted as a true health professional by other health professionals	.041	.626	.042	.036	.018	.196	.064
	Disagreeing with co-workers concerning the treatment of a patient	.199	.590	.313	-.007	.240	.156	-.099
Role/work overload	Having so much work to do that everything cannot be done well	.087	.115	.751	.159	.150	.096	.037
	Not having enough staff to adequately provide necessary services	.155	-.020	.742	.141	.027	-.085	.115
	Keeping up with new developments in order to maintain professional competence	.032	.189	.660	.224	.129	.115	-.123
	Trying to meet society’s expectations for high-quality care	.013	.125	.598	-.150	.236	-.171	.297
	Allowing personal feelings to interfere with the care of patients	.131	.244	.595	.378	.080	.245	-.025
	Being interrupted by people while performing job duties	.370	.012	.541	.184	-.033	.018	.103
	Not being challenged by your work	.360	.204	.531	.210	.036	.161	.022
Conflicts with patients	Dealing with “difficult” patients	.113	.043	.153	.763	.145	.016	.058
	Fearing that a mistake will be made in the treatment of a patient	.078	.081	.211	.752	.078	.037	.040
	Being inadequately prepared to meet the needs of patients	.199	.074	.243	.620	.169	.146	-.021
	Caring for terminally ill patients	.189	.130	-.004	.501	-.009	-.458	.068
	Caring for the emotional needs of patients	.216	.148	.108	.489	.429	.175	-.138
Receiving respect from the patients	Not receiving the respect that you deserve from the general public	.245	.182	.113	.123	.821	.089	.062
	Being uncertain about what to tell a family about a patient’s condition	.165	.105	.171	.247	.803	.160	.052
	Feeling ultimately responsible for patient outcomes	.126	.209	.215	.393	.437	-.214	-.114
Job advancement	Feeling that you are inadequately paid as a health professional	.178	.198	.035	.118	.060	.673	.207
	Feeling that opportunities for advancement on the job are poor	.274	.234	.114	.063	.166	.658	-.001
Work-family conflicts	Having job duties that conflict with family responsibilities	.094	.186	.243	.055	.019	.209	.751

#### Multiple regressions

To examine the effects of friendship network pattern and friendship network supports on job stress, multiple regressions was performed after controlling for any other influence factors. Among the network variables friends from work and emotional support variables were omitted from this model because it had no significant statistics. The sociodemographic characteristics (age, gender, marital status and total household income) of participants were used as control variables. Subjects were divided into three groups by job positions: nurses, doctors and technologists. Network patterns and network supports, among the three groups, are compared to explain the mechanisms giving a positive influence on job stress.

All statistical analyzes were conducted with the STATA12.0 program.

## Results

### General characteristics of participants

Table 2 presents the general characteristics of participants. Of the 420 participants, 288 (68.6%) were nurses, 68 (16.2%) were doctors, and 64 (15.2%) were technologists. The female respondents included 269 (93.4%) nurses, 25 (36.8%) doctors, and 11 (17.2%) technologists. The level of education for the 420 participants was as follows: nurses 68% college and above, doctors 100% college and above and technologists 51% college and above (p = .000). Regarding contribution to household income, 42.9% of technologists were the sole source of their household’s income, which was the highest proportion of the three job positions. However, only 12.5% of the high-income groups (those earning more than 7 million won per month—currency rate 2011, one million won = $1,000) were technologists.

**Table 2:** Characteristics of study population and friendship networks (N = 420) n (%).

	Nurse		Doctor		Radio. Tech		* p-value
	(n = 288)		(n = 68)		(n = 64)		
**Demographic characteristics:**							
**Gender:**							
Female	269	(93.4)	25	(36.8)	11	(17.2)	0.000
**Age (yr):**							
20–29	89	(30.9)	36	(52.9)	25	(39.1)	0.002
30–39	140	(48.6)	24	(35.3)	16	(25.0)	0.000
40–49	54	(18.8)	7	(10.3)	16	(25.0)	0.088
50–59	5	(1.7)	1	(1.5)	7	(10.9)	0.000
**Marital Status:**							
Married	138	(47.9)	26	(38.2)	38	(59.4)	0.052
**Education:**							
Junior college	63	(21.9)	-	-	31	(48.4)	0.000
College	180	(62.5)	50	(73.5)	27	(42.2)	0.000
Graduate school	45	(15.6)	18	(26.5)	6	(9.4)	0.024
**Contribution to household income:**							
100%	73	(25.3)	18	(26.5)	27	(42.9)	0.024
50–99%	114	(39.6)	23	(33.8)	23	(36.5)	0.674
<50%	101	(35.1)	27	(39.7)	13	(20.6)	0.039
**Household income (won):**							
More than 7 million/m	89	(30.9)	36	(52.9)	8	(12.5)	0.000
**Friendship network characteristics:**							
**mean # of friends	10		11		11		
***Friendship network:							
Mean # of friends by types:							
Friends from work	2.3		1.3		1.3		0.000
Friends from college	1.5		2.0		1.2		0.010
Friends from pre-college school^1)^	1.6		1.7		2.1		0.039
Frequency of meeting friends:							
Strong ties (every day)	102	(35.4)	29	(42.6)	22	(34.4)	0.502
Density:							
Acquaintances within friends	257	(89.2)	58	(85.3)	54	(84.4)	0.436
Acquaintances between the two^2)^	276	(95.8)	64	(94.1)	56	(87.5)	0.034
Group activities:							
Alumni reunions inside work	105	(36.5)	18	(26.5)	34	(53.1)	0.006
Alumni reunions outside of work	122	(42.4)	31	(45.6)	42	(65.6)	0.003
**Network supports:**							
Instrumental support	88	(30.6)	17	(25.0)	21	(32.8)	0.581
Life information	173	(60.1)	33	(48.5)	29	(45.3)	0.040
Information about job vacancies	37	(12.8)	10	(14.7)	7	(10.9)	0.813

* p-value according to oneway ANOVA

** Total network size

*** Based on those who self-reported up to seven close friends.

^1)^ High school, middle school, and elementary school,

^2)^ Friends and family

### Friendship network patterns and supports

The average size of friendship networks was 10.8 (Table 2). One would expect that people in the same profession would have a greater affinity for one another, and we assumed that friends who attended college and worked together would have the same job position (64.4% of doctors’ friends, 63.5% of nurses’ friends, and 50% of technologists’ friends were of the same job position). Indeed, we observed that people who were similar to one another tended to get along. In comparison to the job position the friends by type were different (p <. 05). Nurses made more friends at work (mean # of friends, 2.3), doctors made more friends in college (2.0) and technologists made more friends in pre-college schools (2.1). Group activities showed that technologists were the highest percentage of attending alumni reunions; outside work (65.6) and inside work (53.1). Doctors followed up attending their alumni reunions; outside work (45.6). Among Network supports, life information support was the highest percentage of nurses (60.1), doctors (48.5) and technologists (45.3).

### Sources of job stress by occupation

The main findings are presented in Table 3 (three dimensions: organizational setting, job demands and individual setting). The average levels of overall job stress were nurses 52.4, doctors 53.6 and technologists 45.4 (p = .006). Significant differences were found among the job positions regarding the sources of job stress. Three visibly distinct characteristics were as follows: first was organizational settings, no significant difference; second was job demands, role/work overload (p = .000)—conflicts with patients (p = .003)—and receiving respect from patients (p = .006) were the major sources of job stress for both nurses and doctors; third was individual settings, job advancement factor for technologists. Technologists were lower in all individual factors of job stress except job advancement where technologists ranked the highest (p = .1). Technologists slightly ranked higher than doctors in the factor of work-family conflicts. In summary, the organizational setting had little difference, job demands nurses and doctors were found to have the highest job stress and individual setting job advancement factor was the single factor when the technologists were ranked the highest job stress.

**Table 3:** Comparisons of job stress by job position.

Dimensions	organizational setting		Job demands			Individual setting		Total
Factors	Decision-making authority	Conflicts with co-workers	Role/work overload	Conflicts with Patients	Receiving respect from patients	Job advancement	Work-family Conflicts	
Nurse	1.59	1.36	2.06	2.09	1.94	1.31	1.27	52.35
	(4.580)	(4.240)	(5.394)	(3.645)	(2.507)	(1.597)	(1.190)	(16.727)
Doctor	1.71	1.30	2.22	2.13	1.91	1.22	1.10	53.56
	(4.394)	(4.096)	(4.802)	(3.299)	(2.556)	(1.748)	(1.317)	(15.291)
Radiological technologist	1.42	1.31	1.57	1.77	1.63	1.52	1.22	45.38
	(4.761)	(5.808)	(5.145)	(3.836)	(2.161)	(2.426)	(1.061)	(18.509)
*p-value	.093	.791	.000	.003	.006	.122	.590	.006

* p-value according to one-way ANOVA test

### The results of multiple regression models

#### Individual-level attributes

Table 4 shows that contributors to job stress differed by individual characteristics and by job position. As mentioned above, most doctors and technologists in our sample were male, and most nurses in our sample were female. The level of job stress for male doctors was 12.8 higher than female doctors (p < .001). Among the three job positions, the level of job stress for married people was higher than unmarried people as follows; nurse (4.46), doctor (11.60) and technologists (6.46), (p < .05).

**Table 4:** Effects of friendship network patterns and supports.

	Nurse		Doctor		Radiologic Technologist	
	Coefficient	SE	Coefficient	SE	Coefficient	SE
Age	-0.404 **	(0.20)	-0.781**	(0.45)	0.186	(0.37)
Male (ref. female)	3.219	(4.09)	12.839****	(3.75)	-6.481	(6.39)
Married (ref. unmarried)	4.464**	(2.47)	11.599**	(5.50)	6.455**	(6.50)
Household income: >7 million/m (ref. less)	-1.072	(2.21)	-0.776	(3.93)	-16.600***	(6.42)
**Friendship network patterns:**						
**Strength:**						
Strong tie(meet every day)	8.003*	(5.00)	9.281	(9.93)	17.614*	(10.88)
Having friends from college	3.220	(2.70)	2.260	(6.35)	13.849**	(6.54)
Having friend from pre-college school	-4.847**	(2.82)	10.548*	(6.86)	10.096	(8.28)
Strong tie **×** Having friends from college^1)^	-9.306**	(4.50)	-5.650	(10.11)	-3.295	(9.62)
Strong tie **×** Having friends from school^1)^	-1.862	(4.64)	-4.324	(9.74)	-29.041***	(11.22)
**Density:**						
Mutual acquaintance between friends	-8.274***	(3.20)	7.685	(6.54)	-14.576*	(9.06)
Mutual acquaintance between friends and family	-1.556	(5.08)	-12.453	(12.13)	-7.439	(8.63)
**Group activities:**						
Alumni reunions inside workplace	1.161	(2.51)	8.414*	(5.21)	5.610	(5.25)
Alumni reunions outside of workplace	-3.923*	(2.40)	-9.393**	(4.38)	3.840	(5.45)
**Friendship network supports:**						
Instrumental support	0.495	(2.26)	9.141**	(4.72)	-9.218**	(4.85)
Life information	-4.123**	(2.19)	-3.825	(4.42)	-7.963**	(4.58)
Information about job vacancies	7.224**	(3.16)	11.247**	(6.00)	5.070	(6.79)
Constant	76.570****	(8.28)	60.717****	(13.53)	47.022****	(12.29)
N	288		68		64	
R^2^	0.113		0.375		0.520	
F	2.17***		1.91**		3.19****	

* p < 0.1(two-tailed test)

** p < 0.05(two-tailed test)

***p < 0.01(two-tailed test)

**** p < 0.001(two-tailed test)

^1)^ interaction term.

In addition, it is hardly surprising that the technologists with the lowest income (less than 7 million won per month) scored 16.6 points higher (p < .01) on the HPSI than did those who earned more than 7 million, as financial factors have long been recognized as contributors to job satisfaction or job stress.

#### Effects of friendship networks

Effects of friendship network patterns differed among the three job positions (Table 4). The first dimension is strength: After controlling for individual characteristics, the area of friends with strong ties (meet daily) surprisingly showed higher stress compared to meeting people with weak ties (not meet daily); nurses 8.0 points (p < .1). One significant factor showed that nurses with college friends having strong ties showed lower stress of 1.3 (i.e., 9.306–8.003) points on HPSI than did those with weak ties. Friends with strong ties (meet daily) surprisingly showed higher stress for technologists of 17.6 points (p < .1). One significant factor showed that technologists with friends from pre-college schools having strong ties showed lower stress of 11.4 (i.e., 29.041–17.614) points on HPSI than did those with weak ties. Nurses with friends from pre-college schools (regardless of strong or weak ties) lower stress of 4.8 points (p < .05) than those who did not. Contrary to predictions, technologists with friends from college higher stress of 13.8 points (p < .05) than those who did not and doctors with friends from pre-college schools higher stress of 10.5 points (p < .1) than those who did not.

The second dimension is density: Nurses with a high-density friendship network had a lower stress level of 8.3 points (p < .01) than those who did not. Technologists with a high-density friendship network had a lower stress level of 14.6 points (p < .1) than those who did not.

Also, we included two variables under friendship network patterns to address group activities. Doctors who participated in work alumni meetings scored 8.4 points (p < .1) higher on the HPSI than those who did not. However, those who participated in alumni meetings outside the workplace showed the opposite tendency, scoring 9.4 points (p < .05) lower than those who did not. Also, nurses who participated in alumni meetings outside the workplace showed scoring 3.9 points (p < .1) lower than those who did not.

#### Friendship network supports

Finally, we explored the mechanism of friendship network and confirmed that support from friendship networks affects job stress. Technologists who received instrumental support from friends scored 9.2 points (p < .05) lower than did those did not receive such support. However, doctors who received instrumental support from friends scored 9.1 points (p < .05) higher than those who did not. It is hard to interpret the results concerning the doctors because it does not fit our predictions. Nurses who received life information from friends scored 4.1 points (p < .05) lower than did those who did not. Technologists who received life information from friends scored 8.0 points (p < .05) lower than did those who did not. Nurses and doctors who received job-vacancy information experienced higher levels of stress than those who did not (means the difference between groups = 7.2, p < .05 and means the difference between groups = 11.2, p < .05, for nurses and doctors, respectively). That is, nurses and doctors who received job-vacancy information reported high levels of job stress related to their intention to leave their current position. In this case, we assume reverse causality.

## Discussion

Although friendship networks have been associated with better job performance and lower rates of job stress, little empirical attention has been paid to the association between friendship networks and job stress. A strongly connected friendship network provides a pathway for information to flow [61, 62]. This research confirmed that a friendship network is a positive source to reduce job stress after controlling all other covariates.

### Existence of friendship networks effect

This study confirmed the effects of two dimensions of friendship networks (network strength and density) on job stress after controlling for socio-economic variables. Burt (1992) found that persons with networks that supply various types of information and support are promoted earlier than expected [63]. Regarding network strength, those with long-term friendships reported lower levels of job stress only if their ties to their friends were strong. We confirmed the positive effects of job stress of having close friends with whom one meets an average of at least once per day, whereas simply having long-term friendships did not exert this effect. Therefore, both long-term friendships and strong tie with friends are needed to produce positive effects on job stress, but the network patterns varied with job position. First, stress related to job demands was high for nurse [64]. However, nurses who have a friendship network of high density in the workplace had a lower level of stress. Second, technologists are uniquely opposite from nurses in that their workplace friends are harder to develop while their friendship network developed in pre-college schools is irreplaceable. That is, technologists who are emotionally connected with long-term friends and meet with them more frequently outside the workplace have lower levels of job stress.

Second, in terms of network density, social cohesion generally facilitated the transfer of knowledge, and it exerts this effect over and above that exerted by the strength of the tie between two people [65]. Nurses and technologists with friends and those whose friends were acquainted with one another reported less job stress than others. Thus, we confirmed the positive effect of social cohesion on job stress. Interestingly, among doctors, participating in alumni meetings within the workplace increased job stress, whereas participating in alumni meetings outside the workplace had the opposite effect. However, we were unable to interpret these results, and future studies should examine this issue more carefully.

### Friendship network supports

The results also show that life information provided a positive effect on job stress for all job positions. Instrumental support did not show any visible influence. We need to remind that job stress is associated with not only professional but also private lives. Therefore, the efforts to reform working conditions using organization focused strategies are unlikely to reduce stress effectively. A combination of individual and organizational level is often the most useful approach for alleviating stress at the workplace [66, 67]. Nurses and doctors who received job-vacancy information experienced higher levels of stress than those who did not. It is not surprising that the subjects with greater job stress more likely to have the profession [68]. In this case, we assume reverse causality. Instrumental support did not show any visible influence.

The current study has several limitations. Generalization of our findings to the larger population should be done with caution because this study was based on data from only two general hospitals. Despite the homogeneity of the sample, a result of all respondents’ working for the same foundation, considerable variation in job stress was observed among professions. Previous studies have found that gender [69–72] affected the degree of stress experienced by workers. Our regression model explained only 11.3% of the variance in the job stress experienced by nurses, whereas this model was, better able to account for variations in the job stress experienced by doctors and technologists (37.5% and 52.0%, respectively). Although we controlled for individual-level variables, gender influenced the level of job stress due to the gender imbalance in the sample. For example, a research showed that job satisfaction of the working women of banking and education was related to work-life balance [73]. Therefore, future studies should use larger samples.

The second limitation is a sampling bias. In general, individuals that chose not to participate could be individuals with high job stress or people with an inadequate friendship network. If true, then the outcome of this job stress level could be underestimated, or the friendship network could be overestimated. To minimize this sampling error, the largest numbers of clusters were included. Still there were a large number of individuals (especially doctors) in the clinical-specialty groups who declined to participate compared with the small number of individuals who did participate could have led to biased results. Last, our cross-sectional design was another limitation, as it precluded explaining the association between friendship networks and job stress. The larger ongoing study from which these baseline data were drawn may provide further insights and clarify the link between friendship networks and job stress in hospital settings. Despite these limitations, the current study confirmed the effects of friendship networks on job stress.

## Conclusion

We found that friendship networks with strong ties have positive effects on job stress. The results also show that life information support from their friendship network lowers the level of job stress. The mechanisms of influence of each job position are different with the sources and levels of job stress. Therefore, the nurse job position is chiefly composed of females the pattern of friendship network is centered on the workplace. The doctor job position has no pattern of friendship network; however, the group activity revealed a positive effect on job stress in alumni meetings outside the workplace. The technologist job position is a pattern of friendship network that is centered on the long-term friendship formed in pre-college schools. Life information from their network of friends provided a positive effect on job stress for all job positions. Instrumental support did not show any visible influence.

## Supporting Information

S1 Text The Health Professions Stress Inventory. (DOCX)
