# Supplementary material for: Effects of Hospital Workers’ Friendship Networks on Job Stress
Source: PLoS One. 2016 Feb 22;11(2):e0149428. doi: 10.1371/journal.pone.0149428 (PMC4763201; doi:10.1371/journal.pone.0149428)
Supplement: S1 Text — (DOCX) [file pone.0149428.s001.docx]

**S1 Text. The Health Professions Stress Inventory**

Effects of Hospital Workers’ Friendship Networks on Job Stress

Greetings

This study examines the impact of the social network of medical service workers on job stress. If you offer your consent, please fill out the questionnaire. If you do not want to participate, please feel free to pass by without doing it. Your answers will only be used to identify the basis for effective social networks of medical service professionals.

Researcher, Sungyae Shin

**I. General information**

Please fill out or check the appropriate blank.

1. Age: years

2. Gender: female ( ), male ( )

3. Marital status: married ( ), not married ( )

4. Education: Junior College ( ), College/University ( ), Graduate School ( )

5. Household composition: Living alone ( ), Living with companion ( )

6. Professional: Nurse ( ), Doctor ( ), Radiologic technologist ( )

7. Duration of service engagement: years months

8. Household income (won)/year:

1. Below 30 million (2) 30~50 (3) 50~70 (4) more than 70 million

9. Contribution to household income ( %)

**II. Friendship network**

This section has questions about your social network. Please fill out or check the appropriate blanks.

1. How many intimate friends do you have? ( )

This study defined a close friend as a person, who comfortably lends/borrows small amounts of money and/or meets with you on birthdays or holidays

Please answer the following questions related to those intimate friends above.

|  | 2.type of friend | 3. kind of support | 4. duration of friendship | 5. frequency of meeting | 6.know my family |
| --- | --- | --- | --- | --- | --- |
|  | ①Friend from work  ②Friend from college  ③Friends from school^*^  ④Other | ①Information about job vacancies  ②Educational information  ③Life information  ④Emotional support  ⑤Instrumental support  ⑥Other | ( )years | ①everyday  ②other | ①yes  ②no |
| 1 |  |  |  |  |  |
| 2 |  |  |  |  |  |
| 3 |  |  |  |  |  |
| 4 |  |  |  |  |  |
| 5 |  |  |  |  |  |
| 6 |  |  |  |  |  |
| 7 |  |  |  |  |  |

*High School, Middle School, and Elementary School.

7. This continues to reflect on those intimate friends you mentioned above: Put a “1” or “2” in the white square that shows the relationship that your friends have with each other. For example; if your first and second friends **do not know each other** then put a “1” in the first square and if your first and third friends **know each other** then put a “2” in the second square.

1. **do not know each other**
2. **know each other**

|  |  | Friend #2 | Friend #3 | Friend #4 | Friend #5 | Friend #6 | Friend #7 |
| --- | --- | --- | --- | --- | --- | --- | --- |
| 1 |  |  |  |  |  |  |  |
| 2 |  |  |  |  |  |  |  |
| 3 |  |  |  |  |  |  |  |
| 4 |  |  |  |  |  |  |  |
| 5 |  |  |  |  |  |  |  |
| 6 |  |  |  |  |  |  |  |
| 7 |  |  |  |  |  |  |  |

8. Do you participate in the following meetings?

| Classification of meeting | No participation | participation |
| --- | --- | --- |
| Meetings with co-workers |  |  |
| Alumni reunions inside workplace |  |  |
| Alumni reunions outside of workplace |  |  |
| Any other meetings ( ) |  |  |

**III. Job stress**

This is related to job stress comments: Please check the box closest to the degree of your own situation.

| no | contents | never | some | average | often | frequently |
| --- | --- | --- | --- | --- | --- | --- |
| 1 | Having so much work to do that everything cannot be done well |  |  |  |  |  |
| 2 | Not having enough staff to adequately provide necessary services |  |  |  |  |  |
| 3 | Not being challenged by your work |  |  |  |  |  |
| 4 | Being interrupted by people while performing job duties |  |  |  |  |  |
| 5 | Feeling that opportunities for advancement on the job are poor |  |  |  |  |  |
| 6 | Fearing that a mistake will be made in the treatment of a patient |  |  |  |  |  |
| 7 | Trying to meet society’s expectations for high-quality care |  |  |  |  |  |
| 8 | Keeping up with new developments in order to maintain professional competence |  |  |  |  |  |
| 9 | Not being able to use abilities to the fullest extent on the job |  |  |  |  |  |
| 10 | Feeling that you are inadequately paid as a health professional |  |  |  |  |  |
| 11 | Possessing inadequate information regarding a patient’s medical condition |  |  |  |  |  |
| 12 | Not being recognized or accepted as a true health professional by other health professionals |  |  |  |  |  |
| 13 | Disagreeing with co-workers concerning the treatment of a patient |  |  |  |  |  |
| 14 | Experiencing conflicts with co-workers |  |  |  |  |  |
| 15 | Allowing personal feelings to interfere with the care of patients |  |  |  |  |  |
| 16 | Not having opportunities to share feelings with co-workers |  |  |  |  |  |
| 17 | Experiencing conflicts with supervisors |  |  |  |  |  |
| 18 | Not knowing what type of job performance is expected |  |  |  |  |  |
| 19 | Supervising the performance of coworkers |  |  |  |  |  |
| 20 | Not being allowed to participate in making decisions |  |  |  |  |  |
| 21 | Having non-health professionals determine the way you must practice your profession |  |  |  |  |  |
| 22 | Not receiving feedback on job performance |  |  |  |  |  |
| 23 | Not receiving the respect that you deserve from the general public |  |  |  |  |  |
| 24 | Caring for the emotional needs of patients |  |  |  |  |  |
| 25 | Caring for terminally ill patients |  |  |  |  |  |
| 26 | Being uncertain about what to tell a family about a patient’s condition |  |  |  |  |  |
| 27 | Feeling ultimately responsible for patient outcomes |  |  |  |  |  |
| 28 | Dealing with “difficult” patients |  |  |  |  |  |
| 29 | Having job duties that conflict with family responsibilities |  |  |  |  |  |
| 30 | Being inadequately prepared to meet the needs of patients |  |  |  |  |  |
